# Flexible coding schemes in dorsomedial prefrontal cortex underlie decision-making during delay discounting

**DOI:** 10.1101/2023.06.15.545101

**Published:** 2023-06-15

**Authors:** Shelby M. White, Mitchell D. Morningstar, Emanuela De Falco, David N. Linsenbardt, Baofeng Ma, Macedonia A. Parks, Cristine L. Czachowski, Christopher C. Lapish

## Abstract

Determining how an agent decides between a small, immediate versus a larger, delayed reward has provided insight into the psychological and neural basis of decision-making. The tendency to excessively discount the value of delayed rewards is thought to reflect deficits in brain regions critical for impulse control such as the prefrontal cortex (PFC). This study tested the hypothesis that dorsomedial PFC (dmPFC) is critically involved in flexibly managing neural representations of strategies that limit impulsive choices. Optogenetic silencing of neurons in the rat dmPFC increased impulsive choices at an 8 sec, but not 4 sec, delay. Neural recordings from dmPFC ensembles revealed that, at the 8-sec delay, the encoding landscape transitions to reflect a deliberative-like process rather than the schema-like processes observed at the 4-sec delay. These findings show that changes in the encoding landscape reflect changes in task demands and that dmPFC is uniquely involved in decisions requiring deliberation.

## INTRODUCTION

Impulsivity is broadly defined as the predisposition to act prematurely without foresight^1,2^ and is a primary feature of several psychiatric conditions; including substance use disorders (SUD) and schizophrenia^1-3^. Delay discounting (DD) is an established behavioral measure of cognitive impulsivity that measures the rate that a temporally delayed reward is devalued^4,5^. Identifying the neural mechanisms that underlie DD has far reaching consequences, from inspiring novel treatment approaches for several psychiatric conditions to understanding decision-making.

Understanding the cognitive underpinnings of impulsivity requires investigating how decision-making changes when rewards are delayed in time. Models that span several levels of decision-making have conceptualized deliberation as a process whereby evidence is accumulated in a ramping-like manner and terminated at the choice^5-7^. As a complicated behavior is learned, strategies can be implemented that limit the need to deliberate between the available options de novo each time choice is made. Strategy use is effective at reducing impulsive choice^5,8,9^. Conversely, individuals rely on deliberation more when choices are difficult^10,11^. Deliberative processes require effort^12^, are slower^12,13^, and require the ability to weigh future choice options via prospection^7,11^. Therefore, determining how strategies can be implemented and potentially disregarded during difficult decisions that require deliberation is critical to understand the neural basis of an impulsive choice.

A failure to use prospection and thereby make decisions without regard for future consequences is referred to as non-planning impulsivity^2,4,14^, which can be differentiated from impulsivity more generally^15^. Conversely, engaging in prospection reduces impulsive choices, which highlights this construct as a potential target for intervention^9,16,17^. Planning refers to policies enacted prior to deciding that will guide the action taken^18^, while prospection refers to mental time travel where one imagines the future in order to pre-experience an event^9,15,19^. Prospection has been formally implemented in DD models where an agent assigns subjective value to choice options via a cognitive search process to mentally imagine the options during deliberation^7^. Therefore, it is necessary to understand how neural networks in vivo implement strategies that may rely on planning and/or prospection in order to limit impulsive choices.

Several studies have examined the role of the rodent dorsal medial prefrontal cortex (dmPFC) in DD with mixed outcomes. Some studies find effects of manipulating this brain region on DD^20-24^, while others do not^25^. There is a strong rationale for examining dmPFC function in DD, as it is broadly implicated as critical for goal-directed decision-making. This includes monitoring the relationship between actions and outcomes^26-28^ and flexibly managing representations of strategies^29-31^. More generally, it is suggested that dmPFC is involved in integrating contextual information to facilitate the representations of goals or task rules^32^. During DD, neural activity in dmPFC has been implicated in signaling the need to change strategy^33-35^ as well as maintaining value representations^36,37^. Moreover, dmPFC has been implicated in the initiation of deliberative sequences^11^, which require prospection in order to assign value to the delayed reward^7^. Finally, rats with several documented alterations in dmPFC function and neurochemistry lack behavioral correlates of strategy and are more impulsive than those that implement strategy^5,38-43^. Taken together, these data motivate the need to identify the computations performed by dmPFC during a DD task to understand impulsivity.

To understand the computations that underlie the high-level, abstract features encoded in dmPFC, it is necessary to examine neural activity at the ensemble level^44-49^. With this approach, dmPFC ensembles have been shown to track the ongoing cognitive demands of a task^50-52^. Specifically, acquiring a new rule^46,53^ or exposure to new context^47,48^ is accompanied by “remapping” at the ensemble level^27,48^. Further, recently developed dimensionality reduction approaches found flexible dynamics in PFC ensembles where linear dynamics transition to rotational dynamics, which was suggested to reflect decision commitment^6,49^.

This study tested the hypothesis that dmPFC is critically involved in flexibly managing neural representations of strategy during DD. This hypothesis was tested in male rats performing a DD task, where neural activity was actuated via optogenetics and measured at the ensemble level via high density neural recordings.

## RESULTS

### Assessment of Planning Behavior and Strategy in Delay Discounting

**Figure 1.**
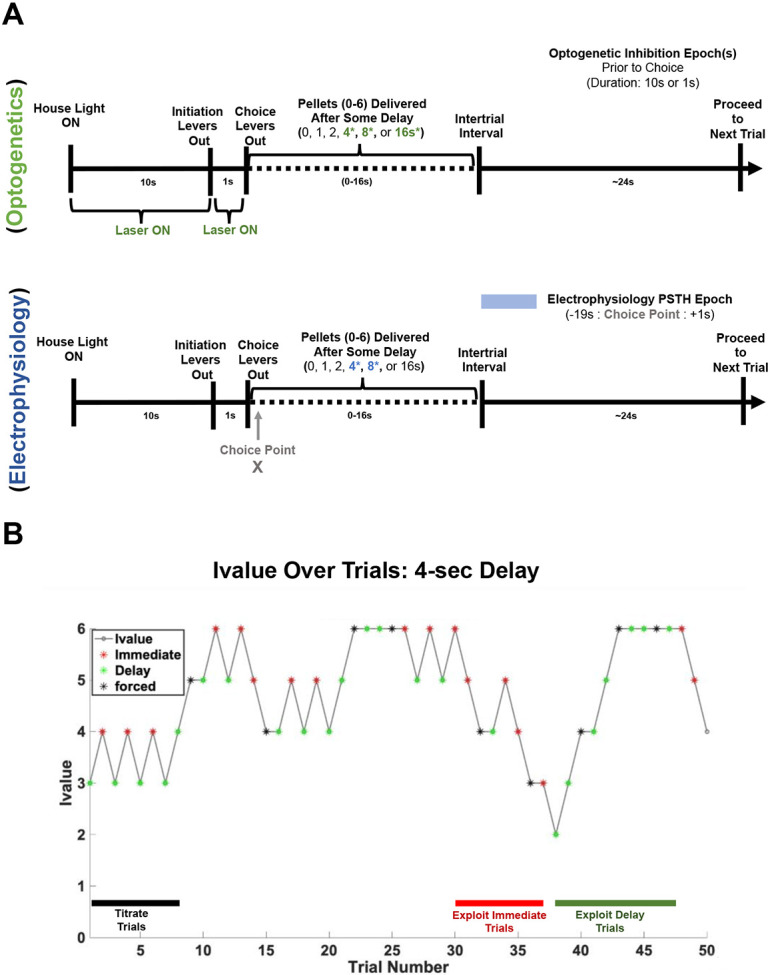
Description of optogenetics and electrophysiology experiments. **A)** Description of single-single trial during DD depicting optogenetic inhibition of dmPFC (**A**, top) and epochs selected for analysis in awake-behaving recordings from dmPFC (**A**, bottom). The green highlighted portion of the trial depicts the points during a single trial where laser was turned ON (**A**, top) while the blue highlighted region (**A**, bottom) depicts the portion of the trial analyzed for the electrophysiology experiment. **B)** Example single-session depicting how an animal makes choices during the 4-sec delay. Different strategies that emerge across trials are also depicted [Immediate-exploit (red), Delay-exploit (green), titration (black)]. Choice trials are depicted in red (immediate choices) and green (delay choices) while forced trials are shown in black. I-value refers to the number of pellets dispensed by the adjusting (Immediate) lever on a given trial.

Optogenetic inhibition and awake-behaving recordings were acquired in separate groups of rats and focused during a period prior to the choice (Figure 1A). Progression of an individual trial is described for optogenetic manipulations (Figure 1A, top) and awake-behaving recordings (Figure 1A, bottom). I-value reflects the number of pellets dispensed by the adjusting lever (Immediate) on a given trial (see methods). The impact of choice on i-value across an individual session is depicted in Figure 1B: Each immediate choice *decreases* and each delay choice *increases* the i-value on the subsequent trial by one. Forced trials have no impact on i-value (Figure 1B). Examples of strategy types (Immediate Exploit, Delay Exploit, titration) are also depicted in Figure 1B.

### Delay choices are associated with correlates of planning behavior

**Figure 2.**
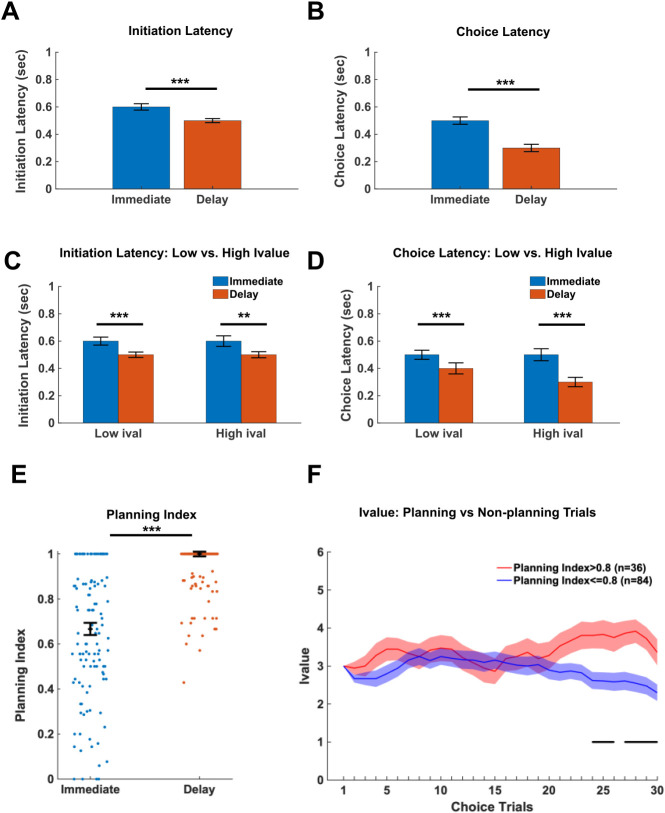
Choices are faster and more consistent when the Delayed option is selected. Assessment of latencies and planning index for optogenetic Laser OFF sessions (4, 8, 16-sec combined; n=8 animals). **A)** Delay initiation latencies (n=1864 choices) are shorter than Immediate (n=1736 choices) latencies (Wilcoxon Rank Sum: *Z*=6.22, *p* =4.98e-10). **B)** Delay choice latencies (n=1864 choices) are shorter than Immediate (n=1736 choices) latencies (Wilcoxon Rank Sum: *Z*=11.59, *p*=4.70e-31). **C)** Initiation latencies are longer for Immediate vs Delay choices for high i-value (>3) choices (Bonferroni-corrected Wilcoxon Rank Sum: *Z*=3.46, *p*=.002; n_Immediate_=899, n_Delay_=852 High i-value choices). Initiation latencies are also longer Immediate choices for low (<=3) i-value choices (Bonferroni-corrected Wilcoxon Rank Sum: *Z*=5.50, *p*=1.13e-7; n_Immediate_=837, n_Delay_=1012 Low i-value choices). **D)** Choice latencies are longer for Immediate vs Delay choices for high i-value (>3) choices (Bonferroni-corrected Wilcoxon Rank Sum: *Z*=7.91, *p*=7.70e-15; n_Immediate_=899, n_Delay_=852 High i-value choices). Choice latencies are also longer Immediate choices for low (<=3) i-value choices (Bonferroni-corrected Wilcoxon Rank Sum: *Z*=8.38, *p*=1.56e-16; n_Immediate_=837, n_Delay_=1012 Low i-value choices). **E)** Planning index during Delay choices was higher than during Immediate choices (Wilcoxon signed-rank test: *Z*=−7.68, *p*=1.57e-14). Individual points show planning index for individual sessions (n=120 sessions) during Immediate or Delay choices. **F)** i-value (Mean +/− SEM) across choice trials was greater for high (red) than low (blue) planning index sessions (Wilcoxon Rank Sum test: *Z*=6.80, *p*=1.06e-11; n=36 high Planning index and n=84 low Planning index sessions). The solid line black line in **F** indicates points during the thirty choice trials where i-value differs for High vs Low Planning Index sessions for FDR-corrected individual Wilcoxon Rank Sum tests at each choice trial (n=30 choice trials). Median latencies (+/− SEM) plotted **A-E.** **P<.01, ***P<.001.

Previous work from our lab suggests that planning is more prevalent on Delay choices than Immediate choices^5^. Planning would be expected to facilitate faster responses, therefore initiation and choice latencies were compared for Immediate and Delay choices in the absence of optogenetic stimulation (Figure 2A-D). Similar to our previous reports, Delay initiation latencies were shorter compared to Immediate (Wilcoxon Rank Sum test *Z*=6.21, *p*=4.98e-10; Figure 2A). In addition, Delay choice latencies were faster than Immediate choice latencies (Wilcoxon Rank Sum test *Z*=11.59, *p*=4.70e-31; Figure 2B). These data indicate that the animals plan Delay choices more than Immediate choices.

Given that the Immediate lever outcome fluctuates in response to previous choices, we investigated whether high (>3) or low (<=3) i-values influence latencies corresponding to Immediate or Delay initiations and choices. In all cases, Delay initiations (Figure 2C) and choices (Figure 2D) were faster than immediate (Bonferroni corrected Wilcoxon Rank Sum tests, *Z’s*>3.46, *p’s*<.01). No differences in initiation latencies were observed for high or low i-value conditions when collapsed across immediate and delay choices (Wilcoxon signed-rank test *Z*=.12, *p* =.90; Figure 2C). Similarly, when comparing choice latencies (Figure 2D), Delay choices were faster than Immediate choices for both low and high i-values (Bonferroni corrected Wilcoxon signed-rank tests *Z’s*>7.90, *p’s*<.001) and no differences were observed between high and low i-value choices when collapsed across Immediate or Delay (Bonferroni corrected Wilcoxon signed-rank test *Z*=−1.83, *p*=.07). Collectively, these results suggest that animals plan their responses on the Delay lever to a greater degree than the Immediate lever irrespective of i-value.

To further assess the hypothesis that Delay choices are planned to a greater degree than Immediate choices, we assessed patterns of responding between the initiation and choice lever for Delay and Immediate choices. For example, consistently using the same lever to both initiate and make a choice (i.e. initiation=Delay lever & choice=Delay lever for a given trial) suggests that the choice is being guided by a pre-existing plan. Alternatively, a minority of animals were observed to consistently initiate with the lever opposite to the choice lever (i.e. initiation on Delay lever and choice on Immediate). To quantify these two different planning types, a planning index was utilized. This value was multiplied by two to keep the range of values between zero (no indication of planning) and one (planning on every trial):

(1) $$\mathrm{Planning}\_\mathrm{index}=2{\;}^{*}\;|\left(\%\;\mathrm{consistent}\;\mathrm{initiation}∕\mathrm{choices}\right)\text{-}0.5)|$$

Providing further support for our hypothesis that animals tend to plan Delay choices to a greater degree than Immediate, a higher planning index was observed for Delay choices (Wilcoxon signed-rank test *Z*=−7.68, *p*=1.57e-14; Figure 2E).

The impact of planning on i-value was also assessed. I-value was higher for high (>.8) planning index compared to low (<=.8) planning index sessions (Wilcoxon Rank Sum test, *Z*=6.80, *p*=1.06e-11; Figure 2F), which was prominent at the end of the session (Figure 2F). Collectively, these results indicate that compared to Immediate choices, animals use a greater degree of planning for Delay choices which corresponds to decreased measures of impulsivity (higher i-value).

### Optogenetic inhibition of dmPFC increases impulsive choices at an 8-sec delay

**Figure 3.**
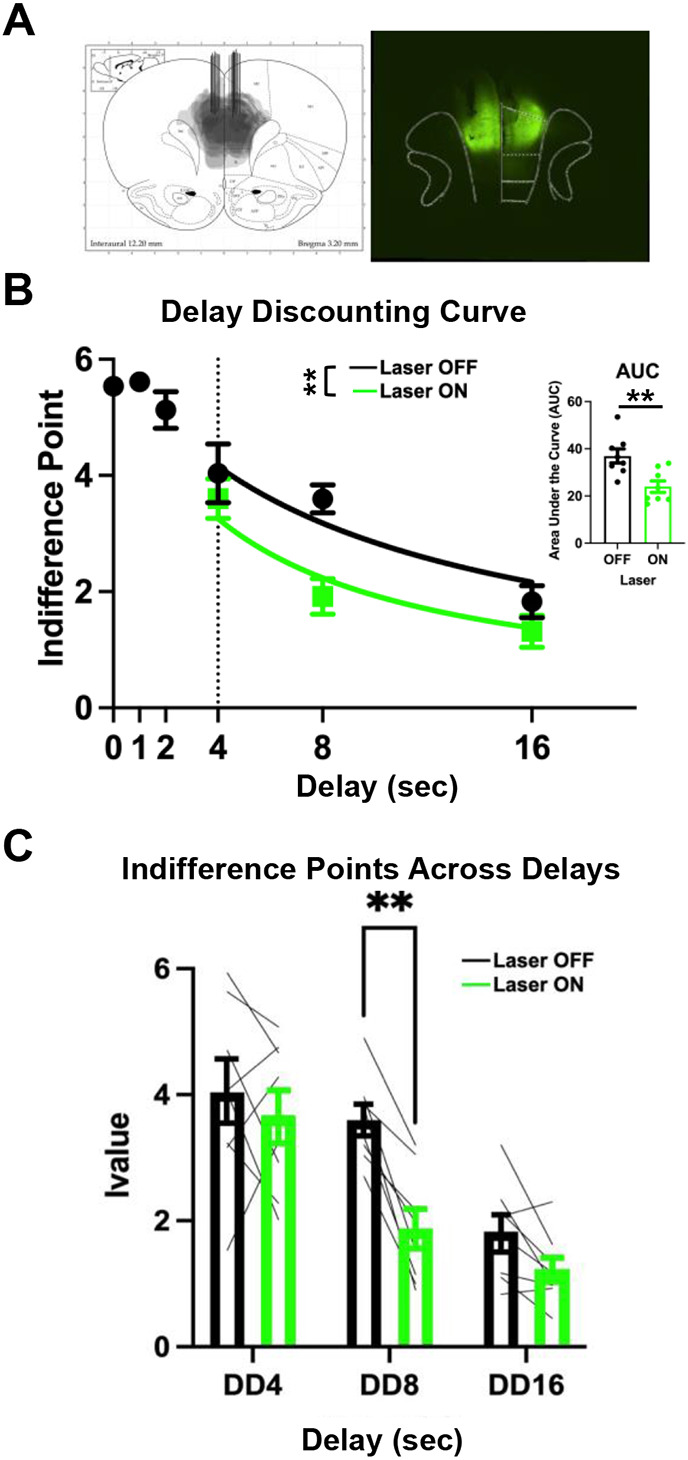
Optogenetic inhibition of dmPFC prior to choice increases impulsivity measures. **(A)** ArchT expression spread and optic fiber placements (left) for all animals (n=8). Representative image of viral spread and optic fiber placements (right). **(B)** Higher DD hyperbolic curve during Laser ON (green) than Laser OFF (black) sessions (4, 8, 16-sec) showing one curve did not adequately account for Laser ON and Laser OFF sessions. Area Under the Curve (AUC) corresponding the DD curve is greater for Laser OFF sessions than Laser ON sessions (paired samples t-test, *t*(7)=5.3, *p*<.01). **(C)** Indifference points decrease as delay increases (ANOVA: F(1.48,10.35)=27.53, p=.0001). Indifference points were decreased on Laser ON (green) sessions compared to Laser OFF (black) sessions (ANOVA: F(1,7)=27.53, p=.003). Specifically, at the 8-sec delay (Holm-Šídák test, *p*<.01) indifference points were decreased for Laser ON (green) compared to Laser OFF (black) sessions but not the 4-sec or 16-sec delays (Holm-Šídák tests, *p’s*>0.05). Optogenetic manipulation occurred at the 4-sec, 8-sec, and 16-sec delays **P< 0.01.

Optogenetic inhibition of dmPFC was used to probe the impact of dmPFC activity on planning and impulsivity. It was hypothesized that inhibiting dmPFC would result in decreased planning, and consequentially, increase measures of impulsivity. Bilateral expression of ArchT in the dmPFC was present for all animals included in the analyses with majority expression in the dorsal mPFC with some ventral spread (Figure 3A). Indifference points for each condition (Laser ON vs Laser OFF) were calculated by averaging the last ten trials of each session for each delay (Figure 3B). A Mazur hyperbolic discounting function^54^ was fit to observations in the Laser ON and Laser OFF conditions (see supplement Figure 1). To assess differences between conditions, direct statistical comparisons of the two discounting curves an Extra-sum-of-squares F-test was used to determine whether one model accurately describes both conditions compared to the individual curves. One curve did not adequately fit both conditions (Extra-sum-of-squares F-test: F(1,46)= 10.46, *p* = .002; Figure 3B), indicating differences in *k*-values between conditions. Specifically, the Laser ON condition resulted in a steeper discounting curve compared to the Laser OFF condition (Figure 3B, left). Additionally, Laser ON vs OFF sessions were compared via Area Under the Curve (Figure 3B, right). Lower AUC for the Laser ON versus OFF was observed (paired samples t-test, *t*(7)=5.3, *p*<.01). Collectively these data indicate that inhibition of the dmPFC increases impulsive responding.

To further assess what drove the differences between the Laser ON and Laser OFF conditions, indifference points were compared at 4, 8, and 16s delays individually (Figure 3C). Greenhouse-Geisser corrected 2-way repeated measures ANOVA revealed a main effect of Delay (F(1.48,10.35)=27.53, p=.0001) and main effect of Laser ON/OFF condition (F(1,7)=27.53, p=.003); Figure 3C). Post hoc comparisons were then used to assess differences in impulsivity at each delay. While no differences were observed between Laser ON versus Laser OFF at the 4 or 16-sec delay (Holm-Sidak tests, *p’s*>0.05), at the 8-sec delay, the indifference point for the Laser OFF condition was larger than Laser ON condition (Holm-Šídák test, *p*<.01; Figure 3C). These results indicate that the indifference points between Laser ON/OFF conditions during the 8-sec delay were the major factor in differences between impulsivity measures. The selective effect at the 8-sec delay may reflect the relatively equal probabilities of choosing either the delay or immediate lever (supplemental Figure 3).

**Figure 4.**
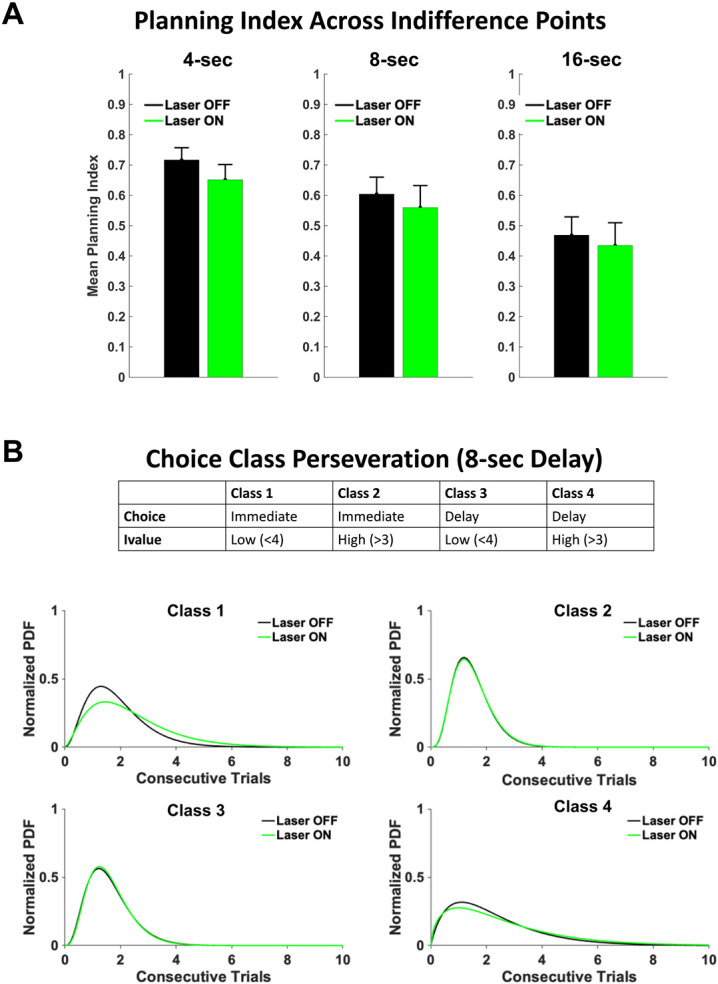
Optogenetic inhibition of dmPFC disrupts strategy change that occurs over trials rather than planning of individual choices. **(A)** Optogenetic inhibition of dmPFC (n=8 animals) did not lower planning index for the 4-sec (*t*(52)=−1.01, *p*=.31), 8-sec (*t*(50)=−0.49, *p*=.63), or 16-sec (*t*(53)=−0.36, *p*=.72) delay. **(B)** Decisions separated into choice classes (B, top) shows optogenetic inhibition increases the number of class 1 choices at the 8-sec delay (Kolmogorov-Smirnov: *p*=0.022).

To investigate the impact of optogenetic inhibition on planning index during the last ten choice trials of the session (indifference points), Laser ON vs Laser OFF conditions were compared across all three experimental delays (4, 8, 16-sec). Contrary to our hypothesis, optogenetic inhibition had no impact on planning index across each delay (Figure 4A). This was true regardless of choice type (Immediate or Delay; data not shown).

The hypothesis was then tested that dmPFC may contribute to strategies that impact choice behavior on longer time scales (i.e., across trials). To examine choice sequences, trials were split into 4 different classes (Figure 4B, top) based on i-value and choice type. This assessed the ability of the animal to deviate from poor choice strategies, such as exploiting the Immediate lever when the i-value is low. Class 1 and Class 4 decisions were considered the least optimal and we hypothesized that optogenetic inhibition would disrupt the ability to switch from exploit strategies in these instances. For both the Laser ON and Laser OFF conditions we determined the distribution of consecutive choices within each class. A gamma-distribution was fit to both the Laser ON and OFF data. Laser ON conditions lengthened consecutive Class 1 decisions in the 8-sec delay (Kolmogorov-Smirnov, p=.022, k=.32; Figure 4B). This indicated that optogenetic inhibition disrupted dmPFC signals required to shift away from poor decisions. No effects of inhibition were seen in the 4-sec delay. Collectively these results suggest that, at the 8-sec delay, optogenetic inhibition of dmPFC increases impulsivity by impacting the animals’ ability to deviate from exploiting the Immediate lever when the value is low.

### dmPFC networks shift from schema encoding at 4-sec to deliberative encoding at 8-sec

**Figure 5.**
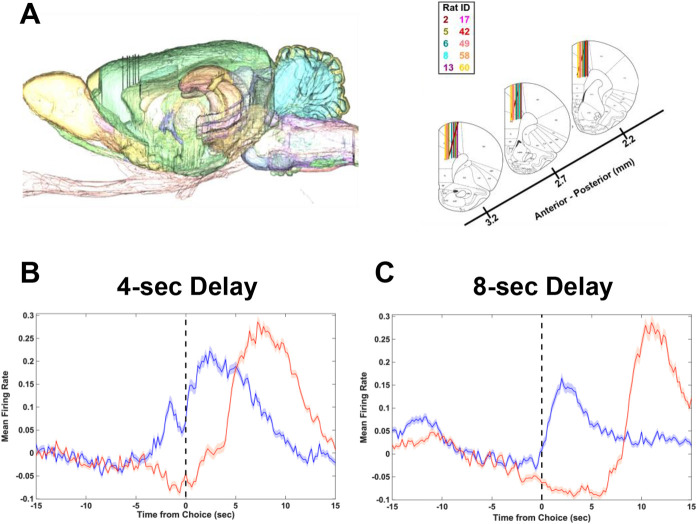
Neural activity of dmPFC neural populations during Immediate and Delay choices during the 4 sec and 8 sec delay. (A) Electrophysiology placements of silicone probes. Representative image of sagittal slice with probe placement in the right dmPFC (left) and for all animals (right). **(B-C)** Grand average mean firing rate for the 4s **(A)** (n=2120 neurons) and 8s **(B)** delay (n=2078 neurons) separated by Immediate (blue) and Delay (red) choices aligned to the time that the animal presses the choice lever (dashed line at time=0).

To determine why optogenetic inhibition of dmPFC increased impulsivity at the 8-sec (but not 4 sec) delay, male Wistar rats were unilaterally implanted with 64-channel silicone probes in dmPFC (see methods; Figure 5A) and recordings during the 4- and 8-sec delay were analyzed. To understand the underlying differences in neural activity patterns for Immediate and Delay choices generally, the mean firing rates for Immediate and Delay choices were aligned to 15-sec prior to the choice and 15-sec after and were plotted for the 4-sec and 8-sec delay. Mean firing rates of choice options exhibited ramping activity prior to the choice point for Immediate and Delay choices and were shifted for 8-sec choices compared to 4-sec choices (Figure 5B, C).

**Figure 6.**
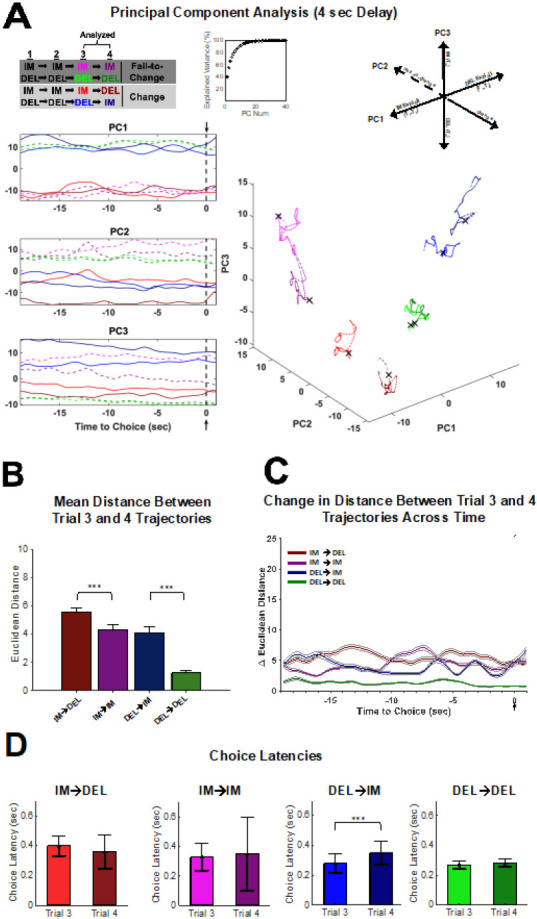
PCA analysis for strategy transitions during the 4-sec delay reveal schema-guided decision-making. **(A)** Population activity (n=581 neurons across n=11 sessions) during the 3^rd^ and 4^th^ trial of a four-trial sequence for all four strategy shifts (IM-Change, IM-Fail-to-Change, DEL-Change, DEL-Fail-to-Change) was analyzed using PCA. Trials 1-3 (T_1-3_) were either IM or DEL exploit trials and Trial 4 (T_4_) was either the ‘Change’ or ‘Fail-to-Change’ trial (see key: A, top left). Top 3 PCs showing trajectories for trial 3 and trial 4 of the four strategy shifts analyzed plotted for each individual top PC (A, left). Dashed lines in the individual PC plots depict Fail-to-Change conditions while solid lines indicate Change conditions (A, left). Cumulative explained variance for the top 40 PCs (A, top middle). Evolution of trajectories for trials 3 and 4 for each strategy condition in 3D PC space for the top 3 PCs, with each dimension corresponding to PC1, PC2, or PC3 (A, bottom right). The **X** denotes the choice point in the trajectory (A, bottom right). Summary of how encoding of task variables relevant to strategy change occurs in 3D PC space (from top 3 PCs), revealing coding schema (A, top right). **(B)** Euclidean distance (mean +/− SEM) between the third and fourth trial trajectory of each strategy shift. The average distance between trials 3 and 4 trajectories did not significantly differ between IM-Fail-to-Change and DEL-Change conditions (Tukey Kramer: *p*=.84) but all other comparisons of strategy shifts were significantly different from one another (Tukey Kramer: *p*<.001). **(C)** Change in Euclidean distance (mean +/− SEM) between trials 3 and 4 across time showing how the distance between trial 3 and 4 differs leading up to the choice point (dashed gray line) for each of the four strategy transitions. PCA conducted on individual sessions for distance measures (B-C; n=11 sessions, see Material and Methods). **(D)** Choice Latencies compared for trials 3 and 4 for each of the four strategy shifts. Choice latencies increase on trial 4 compared to trial 3 only when the animal shifts away from the exploiting the Delay lever (Wilcoxon Signed Rank test: *Z*=−3.74, *p*=.0002). No differences in choice latencies were observed between trial 3 and 4 for any other strategy shift (Wilcoxon signed-rank test: Z’s<1.72, *p*’s>.05). ***P<.001.

Given that optogenetic inhibition occurred prior to the choice point, the remainder of the analyses were conducted on spike trains 19-sec prior to and 1-sec after the choice was made on a given trial (Figure 1A). Additionally, to better understand neural activity related to strategy shifts, the remainder of the analyses focused on trials that occur specifically during exploitation of the Immediate or Delay lever. Immediate and Delay Exploit strategies (Figure 1A) were defined as three or more consecutive choices on the same lever (Immediate or Delay), given that animals would have to continue pressing on the third trial despite being exposed to a forced trial. Therefore, neural activity was assessed between the 3^rd^ and 4^th^ choice trial in the sequence. The 4^th^ choice could either be to continue to exploit (Fail-to-Change strategy) or to shift to the opposite lever (Change strategy). The four types of strategy shifts analyzed were a ‘IM Change’, ‘IM Fail-to-Change’, ‘DEL Change’, and ‘DEL Fail-Change’ strategy (see Figure 6A, top). The four strategy types enabled the assessment of whether neural signatures of continuing to exploit one choice (IM/DEL Fail-Change) differed from those of choosing to abandon an exploit strategy on the fourth trial (IM/DEL Change). To test this, PCA was performed across each strategy type on neurons for the 4-sec (n=581 neurons) and 8-sec (n=1166 neurons) delays to obtain the neural trajectories of the 3^rd^ and 4^th^ trial (see Materials and Methods).

**Figure 7.**
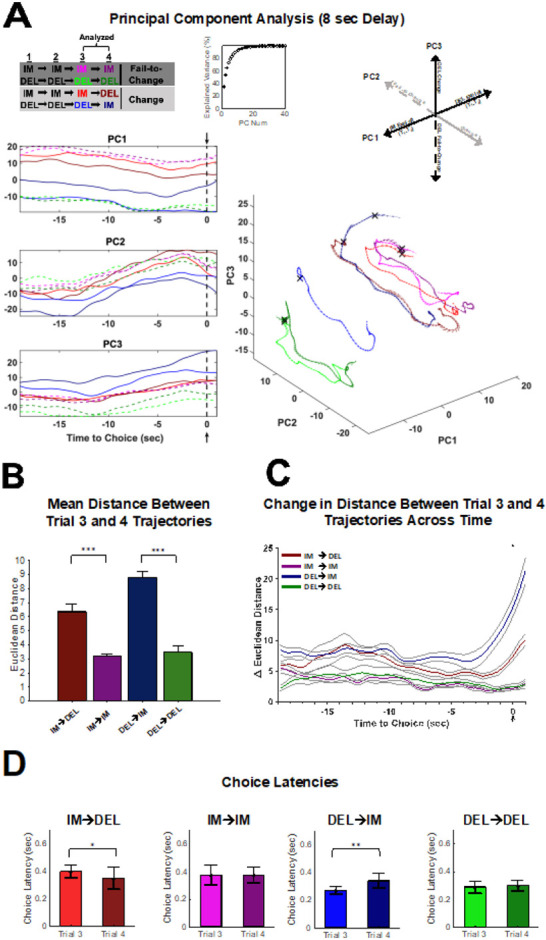
PCA analysis for strategy transitions during the 8-sec delay reveal deliberation-guided decision-making. **(A)** Population activity (n=1166 neurons across n=13 sessions) during the 3^rd^ and 4^th^ trial of a four-trial sequence for all four strategy shifts (IM-Change, IM-Fail-to-Change, DEL-Change, DEL-Fail-to-Change) was analyzed using PCA. Trials 1-3 (T_1-3_) were either IM or DEL exploit trials and Trial 4 (T4) was either the ‘Change’ or ‘Fail-to-Change’ trial (see key: A, top left). Top 3 PCs showing trajectories for trial 3 and trial 4 of the four strategy shifts analyzed plotted for each individual top PC (A, left). Dashed lines in the individual PC plots depict Fail-to-Change conditions while solid lines indicate Change conditions (A, left). Cumulative explained variance for the top 40 PCs (A, top middle). Evolution of how trajectories for trials 3 and 4 for each strategy condition move in 3D PC space for the top 3 PCs, with each dimension corresponding to PC1, PC2, or PC3 (A, bottom right). The **X** denotes the choice point in the trajectory (A, bottom right). Summary of how encoding of task variables relevant to strategy change occurs in 3D PC space (from top 3 PCs), revealing coding schema (A, top right). **(B)** Euclidean distance (mean +/− SEM) between the third and fourth trial trajectory of each strategy shift. The average distance between trials 3 and 4 trajectories did not significantly differ between Fail-to-Change conditions (Tukey Kramer: *p*=.55) but all other comparisons of strategy shifts were significantly different from one another (Tukey Kramer: *p*<.001). **(C)** Change in Euclidean distance (mean +/− SEM) between trials 3 and 4 across time showing how the distance between trial 3 and 4 differs leading up to the choice point (dashed gray line) for each of the four strategy transitions. PCA conducted on individual sessions for distance measures (B-C; n=13 sessions, see Material and Methods). **(D)** Choice Latencies compared for trials 3 and 4 for each of the four strategy shifts. Choice latencies significantly differed between trials 3 and 4 for the DEL-Change (Wilcoxon signed-rank test: *Z*=−3.16, *p* =.002) and IM-Change (Wilcoxon signed-rank test: *Z*=2.37, *p*=.02) strategy conditions. No differences in choice latencies were observed between trial 3 and 4 for the Fail-to-Change strategy conditions (Wilcoxon signed-rank test: *Z*’s<.34, *p*’s>.73). *P<.05, **P<.01, ***P<.001.

The top three PCs and the trajectory of each strategy type are plotted for the 4-sec (Figure 6A) and the 8-sec delays (Figure 7A). For the 4-sec delay, each of the first 3 PC’s clearly separated features of the task (Figure 6A); where PC1 reflected neural activity patterns related to the exploit sequence in which the animal was engaged (IM-IM-IM vs DEL-DEL-DEL), PC2 separated if the animal would continue to exploit or not (Change vs Fail-to-Change), and PC3 separated what choice the animal made on the 4^th^ trial (IM vs DEL). At the 8-sec delay, PC1 still reflected the exploit sequence (Figure 7A). However, PC2 no longer reflected exploit or not, and PC3 reflected DEL-change or DEL-Fail-to-Change sequences (Figure 7A). These data suggest that the encoding schemes in dmPFC differ across the delays of the DD task.

To quantify how encoding differs when an animal continues to exploit vs deviating from an exploit strategy, the mean Euclidean distance between each point in the 3^rd^ trial’s trajectory was compared to each point in the 4^th^ trial’s trajectory for each individual session (Figure 6B, Figure 7B). For both the 4-sec and the 8-sec delay, distances for each Change trial type (e.g. IM→DEL, DEL→IM) were larger than their respective Fail-to-Change types (e.g. IM→IM, DEL→DEL) (Kruskal-Wallis(3,403) *χ*2=270.39, *p*<.001, Figure 6B; Kruskal-Wallis(3,403) *χ*2=304.78 *p*<.001, Figure 7B). This indicates that the neural activity patterns across Change trials 3 and 4 differed more so than Fail-to-Change trials, which may reflect neural activity patterns required to update choice strategy. However, this was common across the 4 and 8-sec delays, therefore difficult to reconcile with the selectivity of the optogenetic inhibition at the 8-sec delay.

The change in distance leading up to the choice between the 3^rd^ and 4^th^ trials was analyzed (Figure 6C and 7C). An interaction between strategy conditions and time was observed in both the 4-sec (Repeated Measures ANOVA F(100,300)=2.956, *p*<.001; Figure 6C) and 8-sec (Repeated Measures ANOVA, F(100,300)=7.130, *p*<.001; Figure 7C) delays. Examination of the temporal patterns of activity captured in the PCs indicated that, at the 4-sec delay, they were relatively stable throughout the trial (Figure 6A). This was quantified in the trajectory differences between the 3^rd^ and 4^th^ trials (Figure 6C) at the 4-sec delay, which did not seem to systematically vary leading up to the choice. The stability of the PCs in time indicates that neural activity encoded something akin to a state variable rather than a dynamic process that might be expected of deliberation preceding a choice. Encoding of a state-like variable was also consistent with the information content that was detected in each PC (Figure 6A).

At the 8-sec delay, neural activity patterns exhibited ramping-like activity leading up to the choice which was most prevalent in PC2 but visually appreciable in each PC (Figure 7A). Ramping-like activity was quantified in the trajectory differences where Change sequences (e.g., IM→DEL, DEL→IM) exhibited ramping-like activity leading up to the choice, while Fail-to-Change trials were static (Figure 7C). These data indicate that, on Change trials, dmPFC neural ensembles exhibit a temporal profile that is consistent with a process like deliberation, where ensembles progressively evolve toward the choice point. This is consistent with the need to deliberate at the 8-sec delay as the probability of choosing either option is roughly equivalent (supplemental Figure 3).

If deliberation was more prevalent at the 8 sec than 4 sec delay, then changes in response latencies should be observed between the 3^rd^ and 4^th^ trials. At the 4-sec delay, animals increased choice latencies between the 3^rd^ and 4^th^ trial for DEL-Change sequences (DEL→IM; Wilcoxon signed-rank test: *Z*=−3.74, *p*=.0002; Figure 6D) but not for Fail-to-Change or IM-Change sequences (Wilcoxon signed-rank tests: *Z*’s<1.72, *p*’s>.05). This indicates that latencies increase only when switching away from the preferred (delay) lever (supplementary Figure 3) at this delay. In contrast, at the 8-sec delay, choice latencies differed between the 3^rd^ and 4^th^ trial for both the IM-Change (Wilcoxon signed-rank test: *Z*=2.37, *p*=.02) and the DEL-Change (Wilcoxon signed-rank test: *Z*=−3.16, *p*=.002) sequences but not for either Fail-to-Change sequences (Wilcoxon signed-rank tests: *Z*’s<.34, *p*’s>.73; Figure 7D). Responses were faster when going from IM→DEL and slower when going from DEL→IM and therefore consistent with the animals either switching to or away from their preferred option, which is consistent with deliberating an easy or difficult choice, respectively. In addition, this suggests that the effect of the optogenetic inhibition at the 8-sec delay interferes with the deliberative process that emerges across dmPFC ensembles.

Theories of DD propose that to evaluate the delay option the magnitude of the reward must be considered in the future context that it will be received. This is hypothesized to rely on a process of “cognitive search” where, as rewards are more temporally delayed, neural trajectories required to arrive at a representation of the value of the future reward are longer. We tested this hypothesis in our data as, if the reward was devalued after 8-sec delay because it was longer, the neural trajectories should cover a larger distance in state-space. This hypothesis was supported, as trajectories at the 8-sec delay were longer than those at the 4-sec delay (Mann-Whitney U test: U=1955, *p*<.001; c.f. Figure 6A, 7A) therefore supporting the observation that deliberative coding scheme is required for the 8-sec but not 4-sec delay.

## DISCUSSION

The main finding of this study is that when a clear choice preference exists (4-sec delay) the neural encoding landscape reflects a schema-driven decision process that changes to a deliberative-driven decision process when a choice preference no longer exists (8-sec delay). This was supported by changes in reaction time when animals update strategies concerning their preferred choice (4-sec delay) or both choice options (8-sec delay). Optogenetic inhibition of dmPFC increased impulsivity measures and increased the number of consecutive low-value choices at the 8-sec delay, suggesting that disrupting the deliberative encoding landscape increases measures of impulsivity by preventing strategy change signals. Collectively these data support the conclusion that the encoding landscape shifts based on the cognitive demands of the task and that impulsive choices are the result of a failure to engage a deliberative process to guide decision-making.

The differences in dmPFC ensemble activity that guide decision-making surrounding strategy change between delays were uncovered by examining PC spaces. When a clear preference for the Delay lever exists (4-sec delay; supplementary Figure 2), neural activity across dmPFC PC spaces were relatively static leading up to the choice. This indicates the existence of a latent signal in dmPFC ensembles that encoded features of the task in a static manner and that existed prior to optogenetic inactivation, which occurred 10 seconds prior to the choice. This may explain the lack of effect of the optogenetic inhibition at the 4-sec delay – any information that dmPFC might contribute to the decision at this delay preceded the inactivation. In addition, a schema encoding strategy may provide a cognitively economical way to choose that limits the need for cognitive resources (e.g., effort, prospection) and facilitates a more procedural way to arrive at a good decision.

In contrast, at the 8-sec delay, PC spaces were more dynamic surrounding strategy changes prior to the choice (i.e., during the window of time that dmPFC was inhibited via optogenetics). These data are consistent with the view that, during DD, dmPFC plays a critical role in signaling the need to change strategy^34^. Our data support and extend this view by indicating that that dmPFC may be uniquely involved when decisions require deliberation.

A number of studies have indicated that dmPFC is critically involved in decision-making when decisions are difficult^33,55^. More deliberation is required for difficult decisions^10,11^ and less deliberation occurs for difficult decisions when dmPFC is inhibited^11^. One explanation for animals failing to change strategy is that optogenetic inhibition of dmPFC disrupted deliberative processes that were more prevalent during the 8-sec delay. Decisions between the Immediate and Delay choice are most difficult at the 8-sec delay as, at this delay, the number of Immediate and Delay choices are roughly equal^5^ (supplementary Figure 2).

The behavior of the trajectories in the PC spaces provide insight into how dmPFC might implement the computations that control decision-making at each delay. While the differences across each delay were robust, a limit to making inferences about these spaces is the shortcoming of PCA. While useful for dimensionality reduction, PCA may not be sufficient to capture dynamics that occur in a high dimensional space given the linear nature of the algorithm^56^. Nonetheless, several important inferences have been made using PCA about latent dynamics that are supported by analysis tools better equipped to describes dynamics in a high-dimensional space.

The differences in the PC spaces across delays suggest that each delay exhibits a different degree of stability. The neural trajectories for each of the trial types at the 4-sec delay were restricted to a discrete region of state space that were generally well-separated from other trial types, suggesting the existence of several meta-stable states (e.g., multistability). Therefore, this suggests that multistability facilitates schema encoding strategies where decision-making is more procedural.

Prior work from our group indicates that neural trajectories in dmPFC track task variables by itinerating through meta-stable states during a foraging-based decision-making task^57,58^ which is reminiscent of the encoding scheme observed at the 4-sec delay. This type of encoding scheme seemed, initially, to be observable at the 8-sec delay. However, at ~12 seconds prior to the choice, PC spaces begin to evolve where PC’s 2 and 3 each begin to move upward (Figure 7A). The change in the way neural trajectories move through state space suggests that a bifurcation may occur and alter the dynamics that control dmPFC networks around this time. After this time, neural trajectories seem to take linear paths that give way to rotational paths near the choice. This type of linear to rotational dynamics is reminiscent of that observed in prefrontal networks of the nonhuman primate, which has been suggested to correspond to decision commitment^6,49^.

Our data support that discounting emerges as a result of a cognitive search process^5,7^. This theory proposes that choice options are evaluated through a process of prospection where options that are ‘easier to find’ are more likely to be chosen and options that are ‘more difficult to find’ are less likely to be chosen. In support of this theory, our data show that the distance between the start of a trajectory and the choice point is shorter (i.e., easier to find) for the 4-sec delay compared to the 8-sec delay regardless of the trial type (Figure 6A, Figure 7A).

The prospective aspect of cognitive search has been conceptualized in the framework of attractor dynamics^7^. Specifically, choice history impacts current choice behavior by imposing a bias that results in decision representations settling into basins of attraction that result in the same choice as what was chosen on the prior trial^59^, possibly because it was ‘easier to find’^7^. Therefore, disrupting dmPFC activity during deliberative processes, where the Immediate and Delay choice options must be weighed, has the potential to negatively impact the ability to ‘find’ the Delay option during prospection. The inability to find the Delay choice option would therefore be hypothesized to result in continued choice of the Immediate option, as was seen with optogenetic inhibition of the dmPFC. This rationale is supported by a recent study that shows that optogenetic *activation* of dmPFC prior to choice during a spatial DD task caused animals to take on a more deliberative rather than procedural strategy^24^, whereas our data show that optogenetic *inhibition* disrupts ability to use deliberative decision-making to implement necessary strategy changes.

Collectively, these observations form the basis of hypotheses that can be directly tested with modern techniques to reconstruct latent dynamics from neural recordings^46,50,52,60^. Specifically, if a bifurcation exists and options are encoded via attractor dynamics, characterizing this will provide insight into how deliberation is implemented in dmPFC networks and how breakdowns in computation responsible for deliberation negatively impact decisions. Identifying methods to repair these computations, such as improving the ability to ‘find’ delay rewards during deliberation could be a powerful approach to reduce impulsivity and thereby improve treatment outcomes in several psychiatric disorders.

## METHODS

Methods and Supplemental materials and any associated references are available in the online version of the paper.

## Supplementary Material

Supplement 1

## References

[1] Dalley J. W., Everitt B. J. & Robbins T. W. Impulsivity, Compulsivity, and Top-Down Cognitive Control. Neuron 69, 680–694 (2011). 21338879 10.1016/j.neuron.2011.01.020

[2] Peterson J. R., Hill C. C., Marshall A. T. & Stuebing S. L. Differences in Impulsive Choice. J. Agric. Food Ind. Organ. 13, 89–99 (2015). 27695664 10.1515/jafio-2015-0024 PMC5045041

[3] Heerey E. A., Robinson B. M., McMahon R. P. & Gold J. M. Delay discounting in schizophrenia. Cogn. Neuropsychiatry 12, 213–221 (2007). 17453902 10.1080/13546800601005900 PMC3746343

[4] Hamilton K. R. Choice impulsivity: definitions, measurement issues, and clinical implications. Personal. Disord. Theory, Res. Treat. 6, 182–198 (2015). 10.1037/per0000099 PMC4535726 25867841

[5] Linsenbardt D. N., Smoker M. P., Janetsian-Fritz S. S. & Lapish C. C. Impulsivity in rodents with a genetic predisposition for excessive alcohol consumption is associated with a lack of a prospective strategy. Cogn. Affect. Behav. Neurosci. 17, 1–17 (2017). 28000083 10.3758/s13415-016-0475-7 PMC5366085

[6] Aoi M. C., Mante V. & Pillow J. W. Prefrontal cortex exhibits multidimensional dynamic encoding during decision-making. Nat. Neurosci. 23, 1410–1420 (2020). 33020653 10.1038/s41593-020-0696-5 PMC7610668

[7] Kurth-Nelson Z., Bickel W. & Redish A. D. A theoretical account of cognitive effects in delay discounting. Eur. J. Neurosci. 35, 1052–1064 (2012). 22487035 10.1111/j.1460-9568.2012.08058.x PMC3774287

[8] Kwan D. Future decision-making without episodic mental time travel. Hippocampus 22, 1215–1219 (2012). 21997930 10.1002/hipo.20981 PMC3262098

[9] Peters J., Bu C., Büchel C., Bu C. & Büchel C. Episodic Future Thinking Reduces Reward Delay Discounting through an Enhancement of Prefrontal-Mediotemporal Interactions. Neuron 66, 138–148 (2010). 20399735 10.1016/j.neuron.2010.03.026

[10] Papale A. E., Stott J. J., Powell N. J., Regier P. S. & Redish A. D. Interactions between deliberation and delay-discounting in rats. Cogn. Affect. Behav. Neurosci. 12, 513–526 (2012). 22588853 10.3758/s13415-012-0097-7 PMC3774285

[11] Schmidt B., Duin A. A. & Redish A. D. Disrupting the medial prefrontal cortex alters hippocampal sequences during deliberative decision making. J. Neurophysiol. 121, 1981–2000 (2019). 30892976 10.1152/jn.00793.2018 PMC6620703

[12] McLaughlin A. E., Diehl G. W. & Redish A. D. Potential roles of the rodent medial prefrontal cortex in conflict resolution between multiple decision-making systems. in International Review of Neurobiology 158, 249–281 (Academic Press, 2021). 33785147 10.1016/bs.irn.2020.11.009 PMC8211383

[13] Van Der Meer M., Kurth-Nelson Z. & Redish A. D. Information processing in decision-making systems. Neuroscientist 18, 342–359 (2012). 22492194 10.1177/1073858411435128 PMC4428660

[14] Bevilacqua L., Goldman D. & Bevilacqua L. Genetics of impulsive behaviour. (2013). 10.1098/rstb.2012.0380 PMC3638385 23440466

[15] Steinberg L. Age Differences in Future Orientation and Delay Discounting. 80, 28–44 (2009). 10.1111/j.1467-8624.2008.01244.x 19236391

[16] Liu L., Feng T., Chen J. & Li H. The Value of Emotion : How Does Episodic Prospection Modulate Delay Discounting ? 8, (2013). 10.1371/journal.pone.0081717 PMC3842935 24312341

[17] O’Donnell S., Oluyomi Daniel T. & Epstein L. H. Does goal relevant episodic future thinking amplify the effect on delay discounting? Conscious. Cogn. 51, 10–16 (2017). 28282631 10.1016/j.concog.2017.02.014 PMC5651988

[18] Ishino S., Takahashi S., Ogawa M. & Sakurai Y. Hippocampal-prefrontal theta phase synchrony in planning of multi-step actions based on memory retrieval. Eur. J. Neurosci. 45, 1313–1324 (2017). 28231381 10.1111/ejn.13547

[19] Atance C. M. & Neill D. K. O. Episodic future thinking. 5, 533–539 (2001). 10.1016/s1364-6613(00)01804-0 11728911

[20] Cardinal R. N., Pennicott D. R., Lakmali C. L., Robbins T. W. & Everitt B. J. Impulsive Choice Induced in Rats by Lesions of the Nucleus Accumbens Core. Science (80-.). 292, 2499–2501 (2001). 10.1126/science.1060818 11375482

[21] Churchwell J. C., Morris A. M., Heurtelou N. M. & Kesner R. P. Interactions Between the Prefrontal Cortex and Amygdala During Delay Discounting and Reversal. Behav. Neurosci. 123, 1185–1196 (2009). 20001103 10.1037/a0017734 PMC2902158

[22] Loos M. Dopamine receptor D1/D5 gene expression in the medial prefrontal cortex predicts impulsive choice in rats. Cereb. Cortex 20, 1064–1070 (2010). 19690230 10.1093/cercor/bhp167

[23] Sonntag K. C. Viral over-expression of D1 dopamine receptors in the prefrontal cortex increase high-risk behaviors in adults: Comparison with adolescents. Psychopharmacology (Berl). 231, 1615–1626 (2014). 24408208 10.1007/s00213-013-3399-8 PMC3969417

[24] McLaughlin A. E. & Redish A. D. Optogenetic disruption of the prelimbic cortex alters long-term decision strategy but not valuation on a spatial delay discounting task. Neurobiol. Learn. Mem. 200, 107734 (2023). 36822467 10.1016/j.nlm.2023.107734 PMC10106449

[25] Feja M. & Koch M. Ventral medial prefrontal cortex inactivation impairs impulse control but does not affect delay-discounting in rats. Behav. Brain Res. 264, 230–239 (2014). 24556205 10.1016/j.bbr.2014.02.013

[26] Francoeur M. J. & Mair R. G. Representation of actions and outcomes in medial prefrontal cortex during delayed conditional decision-making: Population analyses of single neuron activity. Brain Neurosci. Adv. 2, 239821281877386 (2018). 10.1177/2398212818773865 PMC7058214 32166140

[27] Karlsson M. P., Tervo D. G. R. & Karpova A. Y. Network Resets in Medial Prefrontal Cortex Mark the Onset of Behavioral Uncertainty Published by : American Association for the Advancement of Science Linked references are available on JSTOR for this article : Network Resets in Medial Prefrontal Cortex M. 338, 135–139 (2012). 10.1126/science.1226518 23042898

[28] Lim D. H., Yoon Y. J., Her E., Huh S. & Jung M. W. Active maintenance of eligibility trace in rodent prefrontal cortex. Sci. Rep. 10, 1–11 (2020). 33139778 10.1038/s41598-020-75820-0 PMC7608665

[29] Rich E. L. & Shapiro M. Rat Prefrontal Cortical Neurons Selectively Code Strategy Switches. J. Neurosci. 29, 7208–7219 (2009). 19494143 10.1523/JNEUROSCI.6068-08.2009 PMC3229282

[30] Kaefer K., Nardin M., Blahna K. & Csicsvari J. Replay of Behavioral Sequences in the Medial Prefrontal Cortex during Rule Switching. Neuron 106, 154–165.e6 (2020). 32032512 10.1016/j.neuron.2020.01.015

[31] Gisquet-Verrier P. & Delatour B. The role of the rat prelimbic/infralimbic cortex in working memory: Not involved in the short-term maintenance but in monitoring and processing functions. Neuroscience 141, 585–596 (2006). 16713111 10.1016/j.neuroscience.2006.04.009

[32] Euston D. R., Gruber A. J. & Mcnaughton B. L. The Role of Medial Prefrontal Cortex in Memory and Decision Making. Neuron 76, 1057–1070 (2013). 10.1016/j.neuron.2012.12.002 PMC3562704 23259943

[33] Peters G. J., David C. N., Marcus M. D. & Smith D. M. The medial prefrontal cortex is critical for memory retrieval and resolving interference. Learn. Mem. 20, 201–209 (2013). 23512936 10.1101/lm.029249.112 PMC3604648

[34] Powell N. J. & Redish A. D. Representational changes of latent strategies in rat medial prefrontal cortex precede changes in behaviour. Nat. Commun. 7, 1–11 (2016). 10.1038/ncomms12830 PMC5036147 27653278

[35] Schuck N. W. Medial prefrontal cortex predicts internally driven strategy shifts. Neuron 86, 331–340 (2015). 25819613 10.1016/j.neuron.2015.03.015 PMC4425426

[36] Sackett D. A., Moschak T. M. & Carelli R. M. Prelimbic Cortical Neurons Track Preferred Reward Value and Reflect Impulsive Choice during Delay Discounting Behavior. J. Neurosci. 39, 3108–3118 (2019). 30755490 10.1523/JNEUROSCI.2532-18.2019 PMC6468102

[37] Laskowski C. S. The role of the medial prefrontal cortex in updating reward value and avoiding perseveration. Behav. Brain Res. 306, 52–63 (2016). 26965571 10.1016/j.bbr.2016.03.007

[38] Beckwith S. W. & Czachowski C. L. Increased delay discounting tracks with a high ethanol-seeking phenotype and subsequent ethanol seeking but not consumption. Alcohol. Clin. Exp. Res. 38, 2607–2614 (2014). 25335779 10.1111/acer.12523 PMC4251872

[39] De Falco E. Impaired cognitive flexibility and heightened urgency are associated with increased alcohol consumption in rodent models of excessive drinking. Addict. Biol. 26, 1–23 (2021). 10.1111/adb.13004 PMC9219171 33508872

[40] Engleman E. A., Ingraham C. M., McBride W. J., Lumeng L. & Murphy J. M. Extracellular dopamine levels are lower in the medial prefrontal cortex of alcohol-preferring rats compared to Wistar rats. Alcohol 38, 5–12 (2006). 16762687 10.1016/j.alcohol.2006.03.001

[41] Linsenbardt D. N. & Lapish C. C. Neural Firing in the Prefrontal Cortex During Alcohol Intake in Alcohol-Preferring ‘P’ Versus Wistar Rats. Alcohol. Clin. Exp. Res. 39, 1642–1653 (2015). 26250465 10.1111/acer.12804 PMC4558392

[42] Linsenbardt D. N., Timme N. M. & Lapish C. C. Encoding of the intent to drink alcohol by the prefrontal cortex is blunted in rats with a family history of excessive drinking. eNeuro 6, 1–15 (2019). 10.1523/ENEURO.0489-18.2019 PMC6712204 31358511

[43] Timme N. M. Compulsive alcohol drinking in rodents is associated with altered representations of behavioral control and seeking in dorsal medial prefrontal cortex. Nat. Commun. 13, (2022). 10.1038/s41467-022-31731-4 PMC9271071 35810193

[44] Baeg E. H. Dynamics of population code for working memory in the prefrontal cortex. Neuron 40, 177–188 (2003). 14527442 10.1016/s0896-6273(03)00597-x

[45] Christensen A. J., Ott T. & Kepecs A. Cognition and the single neuron: How cell types construct the dynamic computations of frontal cortex. Curr. Opin. Neurobiol. 77, 102630 (2022). 36209695 10.1016/j.conb.2022.102630 PMC10375540

[46] Durstewitz D., Vittoz N. M., Floresco S. B. & Seamans J. K. Abrupt transitions between prefrontal neural ensemble states accompany behavioral transitions during rule learning. Neuron 66, 438–448 (2010). 20471356 10.1016/j.neuron.2010.03.029

[47] Hyman J. M., Ma L., Balaguer-Ballester E., Durstewitz D. & Seamans J. K. Contextual encoding by ensembles of medial prefrontal cortex neurons. Proc. Natl. Acad. Sci. U. S. A. 109, 5086–5091 (2012). 22421138 10.1073/pnas.1114415109 PMC3323965

[48] Ma L., Hyman J. M., Durstewitz D., Phillips A. G. & Seamans J. K. A Quantitative Analysis of Context-Dependent Remapping of Medial Frontal Cortex Neurons and Ensembles. J. Neurosci. 36, 8258–8272 (2016). 27488644 10.1523/JNEUROSCI.3176-15.2016 PMC6601954

[49] Mante V., Sussillo D., Shenoy K. V. & Newsome W. T. Context-dependent computation by recurrent dynamics in prefrontal cortex. Nature 503, 78–84 (2013). 24201281 10.1038/nature12742 PMC4121670

[50] Balaguer-Ballester E., Lapish C. C., Seamans J. K. & Durstewitz D. Attracting dynamics of frontal cortex ensembles during memory-guided decision-making. PLoS Comput. Biol. 7, (2011). 10.1371/journal.pcbi.1002057 PMC3098221 21625577

[51] Mashhoori A., Hashemnia S., McNaughton B. L., Euston D. R. & Gruber A. J. Rat anterior cingulate cortex recalls features of remote reward locations after disfavoured reinforcements. Elife 7, 1–18 (2018). 10.7554/eLife.29793 PMC5931797 29664400

[52] Balaguer-Ballester E., Lapish C. C., Seamans J. K. & Durstewitz D. Attracting states in frontal cortex networks associated with working memory and decision making. BMC Neurosci. 12, 1–2 (2011).21208416

[53] Rigotti M., Rubin D. B. D., Wang X. J. & Fusi S. Internal representation of task rules by recurrent dynamics: The importance of the diversity of neural responses. Front. Comput. Neurosci. 4, 1–29 (2010). 21048899 10.3389/fncom.2010.00024 PMC2967380

[54] Mazur J. E. An adjusting procedure for studying delayed reinforcement. Quant. Anal. Behav. 5, 55–73 (1987).

[55] Peters G. J. & Smith D. M. The Medial Prefrontal Cortex Is Needed for Resolving Interference Even When There Are No Changes in Task Rules and Strategies. Behav. Neurosci. 134, 15–20 (2019). 31789536 10.1037/bne0000347 PMC6944745

[56] Whiteway M. R. & Butts D. A. The quest for interpretable models of neural population activity. Curr. Opin. Neurobiol. 58, 86–93 (2019). 31426024 10.1016/j.conb.2019.07.004

[57] Lapish C. C., Durstewitz D., Chandler L. J. & Seamans J. K. Successful choice behavior is associated with distinct and coherent network states in anterior cingulate cortex. Proc. Natl. Acad. Sci. U. S. A. 105, 11963–11968 (2008). 18708525 10.1073/pnas.0804045105 PMC2516968

[58] De Falco E. The rat medial prefrontal cortex exhibits flexible neural activity states during the performance of an odor span task. eNeuro 6, 1–16 (2019). 10.1523/ENEURO.0424-18.2019 PMC6472939 31008186

[59] Scherbaum S. Process dynamics in delay discounting decisions: An attractor dynamics approach. Judgm. Decis. Mak. 11, 472–495 (2016).

[60] Durstewitz D. A state space approach for piecewise-linear recurrent neural networks for identifying computational dynamics from neural measurements. PLoS Computational Biology 13, (2017). 10.1371/journal.pcbi.1005542 PMC5456035 28574992

[61] Oberlin B. G. & Grahame N. J. High-alcohol preferring mice are more impulsive than low-alcohol preferring mice as measured in the delay discounting task. Alcohol. Clin. Exp. Res. 33, 1294–1303 (2009). 19389183 10.1111/j.1530-0277.2009.00955.x PMC2872785

[62] Pachitariu M., Steinmetz N., Kadir S., Carandini M. & Kenneth D H. Kilosort: realtime spike-sorting for extracellular electrophysiology with hundreds of channels. BioRxiv 61481 (2016).

