# Supplementary material for: Flexible coding schemes in dorsomedial prefrontal cortex underlie decision-making during delay discounting
Source: bioRxiv. 2023 Jun 15:2023.06.15.545101. Preprint. [Version 1] doi: 10.1101/2023.06.15.545101 (PMC10312702; doi:10.1101/2023.06.15.545101)
Supplement: Supplement 1 [file NIHPP2023.06.15.545101v1-supplement-1.pdf]

bioRxiv preprint doi: https://doi.org/10.1101/2023.06.15.545101; this version posted June 15, 2023. The copyright holder for this preprint (which was not certified by peer review) is the author/funder, who has granted bioRxiv a license to display the preprint in perpetuity. It is made available under aCC-BY 4.0 International license.

Supplementary Material

METHODS

Animals

Male Wistar rats were purchased from Envigo (Indianapolis, IN) to complete Experiment 1 (optogenetic inhibition of dmPFC; n=8) and Experiment 2 (awake-behaving electrophysiology in dmPFC; n=10). Following arrival at the vivarium, animals were acclimated for 3 days. A 12-h reverse light/dark cycle with lights off at 7:00 AM was utilized. Following acclimation, animals were single housed and given at least a week prior to testing. All animals were at least 70 days of age prior to testing and had *ad lib* access to food and water prior to food restriction/habituation. Animals were food restricted to 85 % of their starting free-feeding weight and maintained under this condition throughout all experiments except immediately prior to and after surgery. All procedures were approved by the IUPUI School of Science Institutional Animal Care and Use Committee and were in accordance with the National Institutes of Health Guidelines for the Care and Use of Laboratory Animals.

Operant Apparatuses

Eight standard one-compartment operant boxes (20.3cm × 15.9cm × 21.3cm; Med Associates, St Albans, VT) inside of sound attenuating chambers (ENV-018M; MED Associates, St. Albans, VT) were used for both Experiment 1 & 2 Habituation and Shaping protocols. Each box contained left and right retractable levers on one wall, left and right stimulus lights positioned immediately above each lever, and an easily accessible pellet hopper positioned between these left and right positioned devices. The opposite wall contained a house light and a tone generator (2900 Hz) on the topmost position. One custom-built operant box (21.6cm x 25.7cm x 52.0cm) was used to accommodate all electrophysiological experiments. Dimensions, stimuli (including house and cue lights), and retractable levers were all positioned to replicate the conditions of the standard operant boxes as closely as possible. The floor bars of the custom-built box were made of wood polls rather than metal and all metal components of the box were covered in a powder coating to reduce artifacts. In addition, two of the eight standard operant chambers were modified for optogenetic Experiment 1 (see Recording and Stimulation Equipment below for additional information).

Behavioral Procedures

Following single housing, animals were handled daily for a week. Animals were then habituated to the operant chambers in the same manner described in previous work from our group (see Linsenbardt et al., 2016). Following habituation, animals then began the DD procedure.

The within-session adjusting amount DD procedure was a modified version of the procedure performed by Linsenbardt et al. (2017), which was adapted from Oberlin & Grahame (2009) and is illustrated in (see Figure 1A). Stimuli presentation for a given trial is depicted in Figure 1A, for additional detail (see Linsenbardt et al., 2016). Before beginning the DD task, the *immediate* and *delay* lever were assigned for each animal. The “delay lever” was assigned to each animal as their non-preferred side. Side preference was determined during shaping. Choosing the

bioRxiv preprint doi: https://doi.org/10.1101/2023.06.15.545101; this version posted June 15, 2023. The copyright holder for this preprint (which was not certified by peer review) is the author/funder, who has granted bioRxiv a license to display the preprint in perpetuity. It is made available under aCC-BY 4.0 International license.

delay lever always resulted in the delivery of 6 45mg sucrose pellets following a delay (0, 1, 2, 4, 8, or 16-sec). The "immediate lever" was the opposite lever and its value was variable. It began each session with a value of 3 pellets and would increase following delayed choices to a maximum of 6 or decrease following immediate choices to a minimum of 0.

"Forced trials" were implemented for the immediate and delay levers, where two consecutive responses on the same lever would result in a forced trial for the non-chosen lever on the next trial (e.g., trial 1=immediate choice, trial 2=immediate choice, trial 3=delay forced). Implementation of Forced trials is depicted in Figure 1B. If an animal did not lever press for the forced trial, the forced trial would be presented again on subsequent trials until the lever was pressed. The animal had to eventually make a response on the forced trial in order to return to choice trials. There was no effect of forced trials on the value of the immediate lever.

The session terminated either after 30 choice trials or 35 minutes for Experiment 1 (all delays) and for the 0, 1, and 2-sec delays for Experiment 2 (in the standard operant chambers). When Experiment 2 animals were moved from the standard operant boxes to the custom operant box for the awake-behaving recordings (4 and 8-sec delays), sessions terminated after either 40 choice trials or 45min using 20mg sucrose pellets in order to maximize number of trials obtained while recording. The delays were completed in ascending order (0, 1, 2, 4, 8, 16-sec) with a day off in between the start of each new delay. Eight to twelve sessions were given at the 0-sec delay, four sessions at the 1 and 2-sec delay. For Experiment 1, nine sessions were completed at the 4, 8, and 16-sec delays (see supplementary Table 1) to account for optogenetic manipulation. For Experiment 2, recordings during the 4 and 8-s delay were obtained until a viable signal was no longer apparent. Magnitude discrimination was determined at the 0-sec delay in the standard operant chambers using the 45mg sucrose pellets before animals were accepted for surgery with an exclusion criterion of 80% (4.8 pellets) of the maximum reward value (6 pellets) for Experiment 1 and 70% criterion for Experiment 2 (4.2 pellets). The average value of the immediate lever over the last ten choice trials was determined for the last three days of the 0, 1, and 2-sec delay and was used to determine the indifference point of each animal. Animals that were included then either received surgery for Experiment 1 or Experiment 2 (see Surgical Preparation & Implantation for detail).

Indifference points for both Experiment 1 and 2 were obtained by taking the last 10 choice trials of each session for a given delay. For Experiment 1 during 'experimental delays' with optogenetic manipulation (i.e., the 4, 8, and 16-sec delays) the last 10 trials of each day for each condition (No inactivation, Epoch 1 inactivation, and Epoch 2 inactivation) were used to determine an indifference point for each condition at each experimental delay (see supplementary Table 1). Days 1 and 2 were excluded for the No inactivation condition, as animals were becoming familiar with the new delay and indifferences points were not yet stable. Therefore, the last 10 trials of days 4, 6, and 8 were used for calculating indifference points for the No inactivation condition. The last 10 trials of days where Epoch 1 as well as Epoch 2 inactivation occurred were taken for each animal for indifference points on optogenetic manipulation days (see supplementary table 1 for experimental design). The rate of discounting was determined using the Mazur Hyperbolic model (equation 2)[55];

$$v = \frac{a}{1+kd} \qquad \textbf{(2)}$$

Here, *v* represents the subjective value of the reward, *a* is the fixed value of the delay reward (6 pellets), *d* is the length of the delay (4, 8, or 16-sec), and *k* is the value fitted to the hyperbolic function using Least squares regression to the indifference points at the 4, 8, and 16-sec delays.

bioRxiv preprint doi: https://doi.org/10.1101/2023.06.15.545101; this version posted June 15, 2023. The copyright holder for this preprint (which was not certified by peer review) is the author/funder, who has granted bioRxiv a license to display the preprint in perpetuity. It is made available under aCC-BY 4.0 International license.

These delays were the focus of this analysis as no optogenetic manipulation occurred at the 1, or 2-sec delay. The virus was allowed to express for at least three weeks before beginning any optogenetic manipulation.

Surgical Preparation & Implantation

For all surgeries, animals were placed inside a flow box and anaesthetized with isoflurane gas (2% at 4L/h) until sedated, at which point they were placed in a stereotaxic frame and maintained on 0.3-0.5% isoflurane for the duration of the surgery. Artificial tears were then applied. Subsequently, fur was shaved and the skin at the incision site was sanitized with three rounds of both 70% EtOH and betadine before applying a local anesthetic (Marcaine; 5mg/kg s.c.). An anti-inflammatory (Ketofen; 5mg/kg dose s.c.) and antibiotic (Cefazolin; 30mg/kg s.c.) were injected at the nape of the neck (anti-inflammatory and antibiotic) before beginning the incision. Once the skull was exposed and cleaned of blood, bregma-lambda coordinates were identified. Prior to any implantation (probe or optic fiber), four stainless steel anchoring screws were inserted. Following insertion of either Cambridge Probes or optic fibers, a two-compound dental cement was used to adhere implants to anchoring screws. Following completion of surgical procedures for Experiment 1 and 2, animals were maintained in a clean heated cage before being returned to the vivarium.

Opsin Virus Delivery and Implantation of Optic Fibers (Experiment 1)

Two syringe pumps (Pump 11 Elite; Harvard Apparatus, Holliston, MA) were attached to each arm of the stereotaxic frame and loaded with 2μL Hamilton syringes (7002KH, Hamilton Co., Reno, NV). Coordinates for PL dmPFC viral injections occurred at a 20- degree angle and were as follows: +3.2mm AP, +2.0mm ML, -5.2mm DV from Bregma. Holes were drilled into the skull to allow the Hamilton syringes to penetrate the brain tissue. Animals then received bilateral injections of .65μL at a flow rate of .2μL/min of the inhibitory Adeno-associated virus (AAV-CaMKIIa-eArchT3.0-EYFP; K. Deisseroth via UNC Vector Core) followed by 10 minutes of diffusion before retracting the Hamilton syringes. Subsequently, animals received fiber implantation of Dual Fiber-optic cannulas with guiding sockets (DFC_200/245-0.37_3.3mm_GS1.4_FLT; Doric Lenses Inc., Quebec, QC, Canada).

Electrophysiological Probe Implantation (Experiment 2)

A rectangular craniotomy was performed over the right hemisphere of dmPFC (AP: 2.8, ML: 0.3 from bregma) followed by a durotomy and cleaning/hydration of the probe insertion site with a sterile saline solution. Additionally, two ground screws were placed above the cerebellum. A Cambridge Neurotech F (n=5), P (n=4), or E-series (n=1) 64-channel silicon probe on a movable drive (Cambridge Neurotech, Cambridge, UK) was lowered to the target site. Mobility of the movable drive was maintained with a coating of antibiotic ointment.

Stimulation and Recording Equipment

Optogenetic Stimulation

A green (532nm) laser (MGL-FN-532-300mW; Ultralasers Inc., Toronto, Canada) operated through Med Associates Programming via a TTL (Med Associates, St Albans, VT) was utilized for stimulation. From the fiber coupler, a mono patch cord (MFP_200/240/900- 0.22_1m_FC-FC; Doric Lenses Inc., Quebec, QC, Canada) was attached and traversed the sound attenuating chambers terminating at the rotary joint (FRJ_1x1_FC-FC; Doric Lenses) which attached a

bioRxiv preprint doi: https://doi.org/10.1101/2023.06.15.545101; this version posted June 15, 2023. The copyright holder for this preprint (which was not certified by peer review) is the author/funder, who has granted bioRxiv a license to display the preprint in perpetuity. It is made available under aCC-BY 4.0 International license.

Branching Fiberoptic Patchcord (BFP(2)_200/240/ARMO-0.22_0.5m_FCM-GS1.4; Doric Lenses) that was the terminal connection to the animal via guiding socket at the top of the animal's skull. Stimulation did not occur in pulses and remained on for the duration of the epoch to prevent rebound depolarization of cells. Stimulation at the tip of the fiber measured approximately 21mW resulting in predicted irradiance of ~60mW/mm2 at the fiber tip. Larger irradiance values were opted for in order to traverse the entire PL cortex with only one fiber per hemisphere.

Stimulation occurred at one of two different epochs during the task for a given session (Epoch 1 inactivation or Epoch 2 inactivation). Epoch 1 stimulation occurred from the start of a given trial and terminated once an animal initiated the trial (see Figure 1A, top). Stimulation remained on if the animal omitted initiating the trial until a response on an initiation lever was made. Epoch 2 stimulation occurred as soon as the animal initiated a trial and terminated once a choice was made (see Figure 1A, top). Stimulation remained if the choice was omitted until a choice was made on subsequent trials. Stimulation occurred on the third, fifth, seventh, and ninth session/day of the 4, 8, and 16-second delays in order to control for carry over effects of the stimulation as well as to obtain indifference points for the No Inactivation condition. All animals received stimulation at both Epoch 1 and Epoch 2 in a cross-over design (see supplementary table 1) so that half the animals received Epoch 1 on the third and seventh day and Epoch 2 on the fifth and ninth day and the other half of animals received the opposite configuration.

Electrophysiology Equipment

Silicon probes were acquired from Cambridge Neurotech (Cambridge, UK) and interfaced with Omnetics connectors (Omnetics – Minnesota, US). Silicon electrodes were mounted the day prior to surgery to Cambridge Neurotech microdrives. An Intan RHD SPI cable (Intan – CA) connected the headstage to a Doric Commutator (Doric Lenses – Canada) positioned above the operant apparatus. An OpenEphys (OpenEphys – MA) acquisition system was used to collect all electrophysiological data. AnyMaze (ANY-maze Behavioral tracking software – UK) was used to collect all behavioral and locomotor data. ANY-maze locomotor data was synchronized with OpenEphys via an ANY-maze AMI connected to an OpenEphys ADC I/O board. Med PC behavioral events were also synchronized to the electrophysiological recordings via an OpenEphys ADC I/O board. Following sessions with diminished signal, electrodes were lowered 50µm following completion of that session in order to allow any drifting of the probe to occur before the next day's session.

Immunohistochemistry & Histology

Optogenetics (Experiment 1)

Animals were perfused within 14 days after behavioral testing with 4% PFA after receiving a anesthetic dose of urethane (1.5-2.0g/kg). Brains were then fixed in 4% PFA for 24 hours before being placed in a 30% sucrose solution (24-72 hours) and subsequently stored at -20 degrees Celsius until sliced 50 microns thick. In order to assess transduction of glutamatergic pyramidal cells within dmPFC, slices were mounted on gelatin subbed glass slides using an aqueous mounting medium (H-1000-10; Vectashield, Invitrogen). A florescence imaging scope (Nikon Eclipse 80*i*; Melville, NY) was used to verify EYFP-tagged protein expression.

Electrophysiology (Experiment 2)

bioRxiv preprint doi: https://doi.org/10.1101/2023.06.15.545101; this version posted June 15, 2023. The copyright holder for this preprint (which was not certified by peer review) is the author/funder, who has granted bioRxiv a license to display the preprint in perpetuity. It is made available under aCC-BY 4.0 International license.

Animals were anesthetized with urethane (1.5 – 2.0 g/kg) and subsequently perfused following with 4% PFA after cessation of spinal reflexes. Following tissue extraction, brains were fixed in 4% PFA for 24 hours and then transferred to a 30% sucrose solution for cryoprotection. Following our post-fix procedures, tissue was stored at -80 degrees Celsius until Experiment 2's cohort was complete. Tissue was sliced at 50 microns and stained for both GFAP and DAPI. Briefly, tissue sections were washed in phosphate buffered saline (PBS) once. Following this, sections were washed in PBS and 0.1% Triton 100. Sections were blocked in 1% normal goat serum. Following blocking, the primary antibody (GFAP; goat anti-chicken) was added and allowed to incubate while shaking for 24 hours at 4°C. Tissue was washed 3x in PBS and then the secondary antibody was added (Alexa fluor 555; goat anti-chicken). Tissue was incubated and shook in a light-protected box for 2 hours at room temperature. Tissue sections were subsequently handled under light-protective materials. Three additional washes in PBS were followed by the addition of DAPI which was allowed to incubate for 10 minutes at room temperature. Three additional washes in PBS followed. Sections were then mounted on gelatin subbed glass slides with anti-fade mounting medium (sc-516212 Santa Cruz Biotechnology) and imaged in order to confirm placement across the dmPFC. Sections were mounted on gelatin subbed glass slides and then imaged in order to confirm placement across the dmPFC.

Spike sorting

Putative neurons were organized into clusters by Kilosort 2[62]. Following automatic spike-sorting, we determined which of these putative neurons met a qualitative criterion in Phy2 (https://github.com/cortex-lab/phy). Specifically, we ensured that the autocorrelograms contained no refractory violations, we ensured that our waveforms were characteristic of an action potential, and that our signal was minimally contaminated by any noise artifacts. Following qualitative characterization in Phy2, we imported our data into MATLAB (Nantucket, MA) for subsequent analyses. A custom MATLAB routine was used to align spike trains to task events. Spike trains were smoothed using Gaussian convolution with a bin width of 200ms and σ set to 10ms.

Data Analysis and Statistical Procedures

Data were structured using custom MATLAB routines and subsequently analyzed and graphed using MATLAB for both Experiment 1 and 2. Analysis of initiation and choice latencies used medians, as medians are less sensitive to means to the positive skew of reaction times.

Decision classes (class 1-4) were constructed to parse decisions into four categories based off choice (immediate or delay) and i-value (high or low; see Figure 4B, supplementary Figure 1). Distribution of decision classes were analyzed using a probability density function (PDF) for Laser ON vs OFF conditions (Experiment 1; Figure 4B) and electrophysiological animals (Experiment 2; supplementary Figure 1) to determine how consecutive choices are made within each class. Class 1 and 4 were considered disadvantageous and therefore animals should deviate from exploiting the immediate (class 1) or delay lever (class 4).

To better understand how animals deviate from exploitation strategies, three or more consecutive choices on either the immediate or delay lever were defined as either *immediate exploitation* or a *delay exploitation* strategy, respectively (Figure 1B). Three consecutive choices were defined as exploitation given that an animal continued to choose the same lever despite being exposed to the other lever via a forced trial. Determining whether differences in neural activity exist for when animals continued to exploit a choice option (e.g., four choices on the same lever) vs deviate from exploitation (e.g., three consecutive choices followed by a choice

bioRxiv preprint doi: https://doi.org/10.1101/2023.06.15.545101; this version posted June 15, 2023. The copyright holder for this preprint (which was not certified by peer review) is the author/funder, who has granted bioRxiv a license to display the preprint in perpetuity. It is made available under aCC-BY 4.0 International license.

on the opposite lever) were then analyzed using PCA. Continued exploitation of a choice option was defined as either Immediate-Fail-to-Change or Delay-Fail-to-Change whereas deviating from the exploit strategy was defined as either Immediate-Change or Delay-Change, resulting in four strategy conditions to be analyzed (see Figures 6-8).

PCA was run separately for the 4-sec and 8-sec delays. For all PCA analyses, spike trains were aligned to the choice point comprised of an interval of 19 seconds prior to and 1 second after the choice (-19s to +1s). Using the choice-point-aligned spike trains, for each neuron, the firing rates (FR) were normalized by the mean firing rate of that unit. Each PCA analysis consisted of 101 time bins (200ms each bin). To be included in the PCA analysis, only neurons firing in all eight trial types (see Figure 6A key for trial types) that occur during trials three and four of the four possible strategy conditions were included in the analysis. Firing rates were normalized and smoothed using a moving average filter spanning 5 bins for each of the eight trial types (trials three and four for each of the four strategy conditions). The eight vectors corresponding to the eight trial types were then concatenated and z-scored prior to running PCA. FR from each neuron (n=581 4-sec, n=1166 8-sec) were contained in columns and each of the 101 time bins per trial type in rows. For example, the for the 4-sec delay, the matrix F(808 X 581) contained n=581 neurons and each column and rows consisted of eight blocks containing the 101 timepoints for each condition (total of 808). PCA was then conducted to analyzed neural activity across all 101 time bins for trials three and four of each strategy condition. The 3 most explanatory dimensions were chosen (top 3 PCs).

PCA for each individual session (4-sec or 8-sec delay) was conducted to obtain trajectories for each of the eight trial types. Using the trajectory coordinates derived from individual session PCA, the mean Euclidean distance in PCA state-space was calculated between the 3rd and 4th trial of the series for each strategy condition. PCA for the choice classes (1-4) were conducted in the same manner. The only factor that differed in this analysis was that only four trial types were present for each PCA conducted, given that each class only contained one of the possible two choice alternatives (Immediate or Delay). Therefore, class 1-2 PCA contained four trial types (3rd and 4th trials) corresponded to either the Immediate Change or Fail-to-Change strategies, while class 3-4 contained the 3rd and 4th trial corresponding to the Delay Change or Fail-to-Change strategy conditions. The number of neurons used within each PCA analysis is depicted in Supplementary Table 2.

bioRxiv preprint doi: https://doi.org/10.1101/2023.06.15.545101; this version posted June 15, 2023. The copyright holder for this preprint (which was not certified by peer review) is the author/funder, who has granted bioRxiv a license to display the preprint in perpetuity. It is made available under aCC-BY 4.0 International license.

Tables/Figures

	Day 1	Day 2	Day 3	Day 4	Day 5	Day 6	Day 7	Day 8	Day 9
A	X	X	1	X	2	X	1	X	2
B	X	X	2	X	1	X	2	X	1

Table S1: **Schedule of optogenetic inhibition for each experimental delay (4, 8, 16-sec) for Experiment 1.** Each **X** indicates a Laser OFF day. The 1 and 2 indicate Laser ON days (Epoch 1 or 2 inactivation; see Figure 1A). Epochs 1 and 2 were alternated every other day such that each animal received two 'Laser ON days' at both Epoch 1 and 2. See Figure 1 for Epoch 1 and 2 duration prior to choice. Days 1 and 2 were discarded from analyses as animals were adjusting to the new delay and behavior was unstable.

Delay	Starting Neuron Number	Number Excluded Neurons	Total neurons used for PCA	Session Numbers Used for PCA Analysis
4s	2120	1539	581	1, 4, 7, 9, 15, 16, 21, 44, 45, 48, 50
8s	2078	912	1166	23, 28, 29, 30, 31, 32, 43, 52, 53, 54, 55, 57, 58

Table S2. Number of neurons and session numbers used in each PCA analysis. The total neurons, excluded neurons, and end number of neurons are included. Neurons were excluded for not firing in all trial conditions tested in the PCA analysis. See Materials and Methods for exclusion criteria.

bioRxiv preprint doi: https://doi.org/10.1101/2023.06.15.545101; this version posted June 15, 2023. The copyright holder for this preprint (which was not certified by peer review) is the author/funder, who has granted bioRxiv a license to display the preprint in perpetuity. It is made available under aCC-BY 4.0 International license.

Choice Class Perseveration (8-sec Delay)

	Class 1	Class 2	Class 3	Class 4
**Choice**	Immediate	Immediate	Delay	Delay
**Ivalue**	Low (<4)	High (>3)	Low (<4)	High (>3)

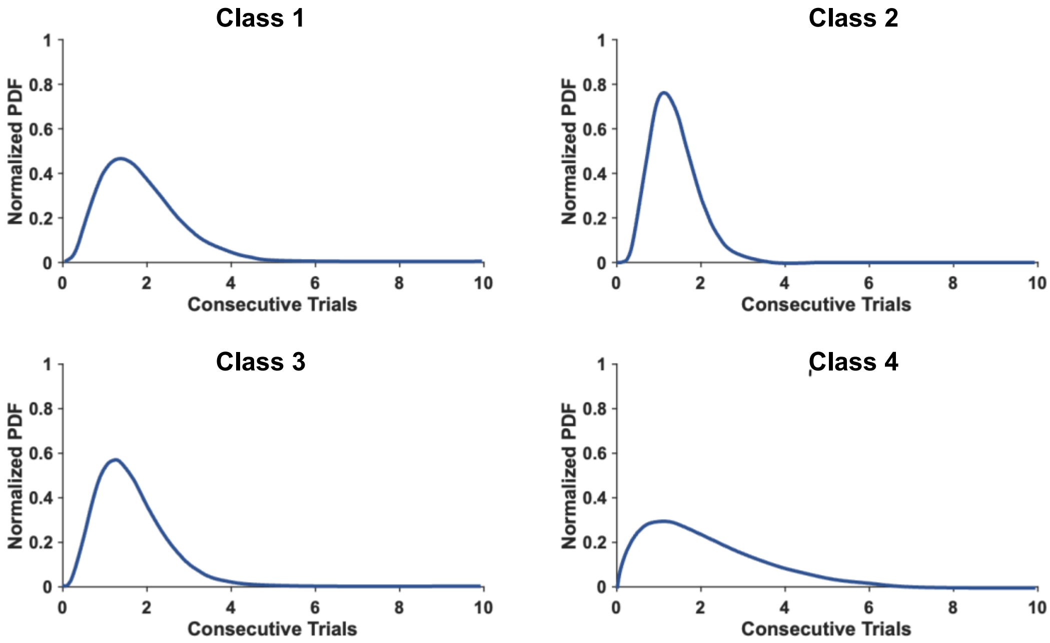

Figure S1. Experiment 2 awake-behaving electrophysiology animals normalized probability density function distribution of consecutive choices for each individual choice class.

bioRxiv preprint doi: https://doi.org/10.1101/2023.06.15.545101; this version posted June 15, 2023. The copyright holder for this preprint (which was not certified by peer review) is the author/funder, who has granted bioRxiv a license to display the preprint in perpetuity. It is made available under aCC-BY 4.0 International license.

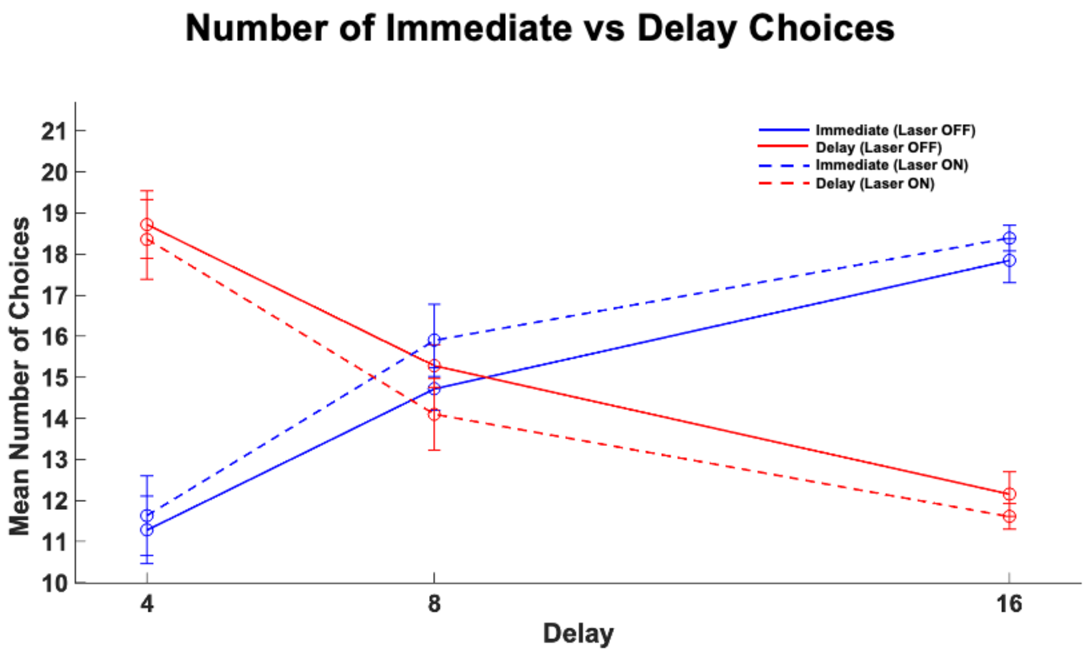

Figure S2. Number of Immediate and Delay choices made during the 4, 8, and 16-sec delays for Experiment 1 optogenetic animals during Laser ON and Laser OFF sessions. Sessions where no optogenetic manipulation (Laser OFF) sessions are depicted using dashed lines while optogenetic manipulation sessions (Laser ON) are depicted by solid lines. Mean and +/- SEM are depicted for each delay. Statistically significant interaction between Choice Lever (IM or DEL) and Delay (2-way ANOVA: $F(2,191) = 52.43$, $p < 0.001$). For Laser OFF sessions, the number of Immediate and Delay choices did not differ at the 8-sec delay ($p > .05$), however the number of Delay choices were greater at the 4-sec delay ($p < .001$) and Immediate choices were more numerous at the 16-sec delay ($p < .001$), as indicated by a Scheffe multiple comparison test.

bioRxiv preprint doi: https://doi.org/10.1101/2023.06.15.545101; this version posted June 15, 2023. The copyright holder for this preprint (which was not certified by peer review) is the author/funder, who has granted bioRxiv a license to display the preprint in perpetuity. It is made available under aCC-BY 4.0 International license.

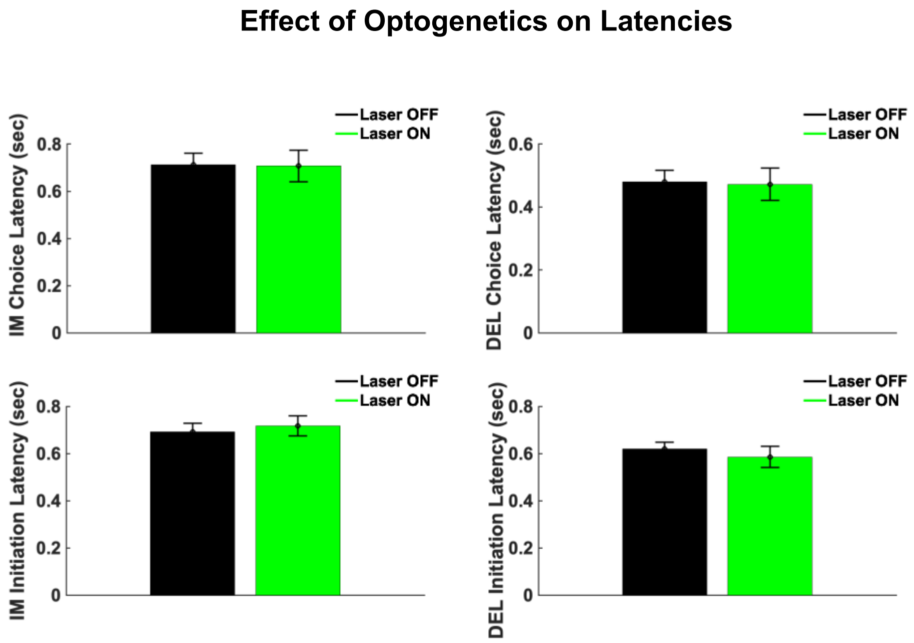

Figure S3. No effect of optogenetic inhibition of dmPFC on initiation or choice latencies (Median +/- SEM) for either Immediate (IM) or Delay (DEL) choices (Wilcoxon Rank Sum test: $Z<.70$, $p>.48$).
